# Comparison of Mid-Term Prognosis in Intermediate-to-Low-Risk Contemporary Population with Guidelines-Oriented Age Cutoff

**DOI:** 10.3390/jcdd11010033

**Published:** 2024-01-22

**Authors:** Stefano Benenati, Francesco Gallo, Won-keun Kim, Arif A. Khokhar, Tobias Zeus, Stefan Toggweiler, Roberto Galea, Federico De Marco, Antonio Mangieri, Damiano Regazzoli, Bernhard Reimers, Luis Nombela-Franco, Marco Barbanti, Ander Regueiro, Tommaso Piva, Josep Rodes-Cabau, Italo Porto, Antonio Colombo, Francesco Giannini, Alessandro Sticchi

**Affiliations:** 1 Dipartimento di Medicina Interna e Specialità Mediche (DIMI), University of Genoa, 16126 Genoa, Italyitalo.porto@gmail.com (I.P.); 2 Interventional Cardiology, Department of Cardio-Thoracic and Vascular Sciences, Ospedale dell’Angelo, AULSS3 Serenissima, Mestre, 30174 Venezia, Italy; 3 Department of Cardiology, Kerckhoff Heart Center, 61231 Bad Nauheim, Germany; dr.won.kim@gmail.com; 4 Cardiology Service, Imperial College Healthcare NHS Trust, London W12 0HS, UK; 5 Division of Cardiology, Pulmonology and Vascular Medicine, Heinrich Heine University, 40225 Duesseldorf, Germany; 6 Department of Cardiology, Cantonal Hospital Lucern, 6000 Luzern, Switzerland; 7 Department of Cardiology, Inselspital, Bern University Hospital, University of Bern, 3010 Bern, Switzerland; roberto.galea@insel.ch; 8 Centro Cardiologico Monzino IRCCS, 20100 Milan, Italy; 9 Cardio Center, IRCCS Humanitas Research Hospital, Rozzano, 20089 Milan, Italydamiano.regazzoli@gmail.com (D.R.);; 10 Department of Biomedical Sciences, Humanitas University, Pieve Emanuele, 20072 Milan, Italy; 11 Interventional Cardiology Unit, Hospital Àlvaro Cunqueiro, 36312 Vigo, Spain; 12 Faculty of Medicine and Surgery, Università degli Studi di Enna “Kore”, 94100 Enna, Italy; 13 Cardiovascular Institute, Hospital Clinic, Institut D’investigacions Biomèdiques August Pi I Sunyer (IDIBAPS), 08036 Barcelona, Spain; 14 Interventional Cardiology, Ospedali Riuniti Di Ancona, 60126 Ancona, Italy; tommaso.piva@ospedaliriuniti.marche.it; 15 Quebec Heart and Lung Institute, Laval University, Quebec City, QC G1V 4G5, Canada; 16 Interventional Cardiology Unit, IRCCS Ospedale Galeazzi Sant’Ambrogio, 20157 Milan, Italy; 17 Cardiac Catheterisation Laboratory, Cardiothoracic and Vascular Department, Azienda Ospedaliero Universitaria Pisana, 56124 Pisa, Italy; 18 Dipartimento di Patologia Chirurgica, University of Pisa, Medica, Molecolare e dell’Area Critica, 56126 Pisa, Italy

**Keywords:** aortic stenosis, transcatheter aortic valve replacement, age, surgical risk, low–intermediate risk

## Abstract

Background: Current European guidelines support transcatheter aortic valve implantation (TAVI) in intermediate-to-low-risk patients ≥75 years-old, but its prognostic relevance is unknown. Methods: Intermediate-to-low-risk (The Society of Thoracic Surgeons score <8%) patients enrolled in the HORSE registry were included. We compared the populations aged under 75 with those over 75. The primary endpoint was all-cause mortality. Results: A total of 2685 patients were included: 280 (8.6%) < 75 and 2405 ≥ 75 years. Through a mean follow-up of 437 ± 381 days, 198 (8.2%) and 23 (8.2%) patients died in the two arms without statistically significant differences (log-rank *p* = 0.925). At Cox regression analysis, age did not predict the occurrence of all-cause death, neither as a continuous variable (HR 1.01, 95% CI 0.99–1.04, *p* = 0.294) nor dichotomizing according to the prespecified cutoff of 75 years (HR 0.97, 95% CI 0.63–1.51, *p* = 0.924). Time-to-event ROC curves showed low accuracy of age to predict all-cause mortality (area under the curve of 0.54 for both 1-year and 2-year outcomes). Conclusions: TAVI has comparable benefits across age strata for intermediate-to-low-risk patients. The age cutoff suggested by the current guidelines is not predictive of the risk of adverse events during hospital stays or of all-cause mortality through a mid-term follow-up.

## 1. Introduction

Transcatheter aortic valve implantation (TAVI) has revolutionized the management of patients with severe aortic stenosis (AS), offering a less invasive alternative to surgical aortic valve replacement (SAVR). Historically, TAVI was primarily reserved for elderly patients who were at high surgical risk due to age-related complications. However, with the advancement of medical technology and extensive research, the surgical risk and age criterion for TAVI candidacy has evolved, challenging the notion that this procedure is mainly dedicated to the very elderly [1,2,3].

Randomized controlled trials (RCTs) have played a pivotal role in establishing the efficacy and safety of TAVI, demonstrating its superiority or non-inferiority compared to SAVR, especially in high- and intermediate-to-low-risk patients. These trials have showcased excellent procedural success and valve performance in individuals below the age previously considered for TAVI. Consequently, the age-old adage of limiting TAVI to the very elderly has become obsolete [1,2,3,4,5,6,7,8].

In 2021, the European Society of Cardiology (ESC)/European Association for Cardio-Thoracic Surgery (EACTS) guidelines introduced a significant paradigm shift. The guidelines recommended transfemoral TAVI with a Class I, Level of Evidence A (IA) rating for patients at a high surgical risk or those aged 75 and above, regardless of their surgical risk. Conversely, a Class IB recommendation was made in favor of SAVR for individuals below the age of 75 and deemed to have a low surgical risk. This age threshold of 75 years emerged as a pivotal determinant, significantly influencing the choice of intervention, particularly in intermediate- and low-surgical-risk patients [9].

However, it is crucial to recognize that while age serves as a convenient demarcation, it is not the sole factor determining the choice of intervention. Dichotomizing patients based solely on age overlooks other critical considerations and has not shown substantial impacts on hard clinical outcomes. Factors such as overall health, comorbidities, and individual patient preferences should be integral components of the decision-making process.

In light of these developments, our study aims to delve deeper into the clinical outcomes of patients below and above the age threshold recommended by the ESC/EACTS guidelines. Specifically, we seek to explore the prognostic relevance of age in intermediate-to-low-risk patients undergoing TAVI with self-expandable devices. By comprehensively analyzing these factors, we aim to provide a nuanced understanding of the interplay between age, procedural outcomes, and long-term prognosis in the context of TAVI, contributing valuable insights to the evolving landscape of aortic valve interventions.

## 2. Materials and Methods

The HORSE registry is an international registry in which patients undergoing transfemoral TAVI using self-expandable valves were retrospectively enrolled across sixteen European centers between September 2014 and April 2020 [10]. All patients provided informed consent to participate in the registry and agreed to the use of their data for scientific purposes.

The research was carried out in accordance with the Declaration of Helsinki (World Medical Association, October 2013) [11]. Criteria for exclusion included pure aortic regurgitation, surgical prosthesis degeneration, and non-transfemoral access. Outcomes were defined according to the Valve Academic Research Consortium 2 criteria [12]. The primary outcome for the present analysis was all-cause mortality. Secondary endpoints included major and minor vascular complications, anulus rupture, new, permanent pacemaker implantation, periprocedural myocardial infarction, cardiac tamponade, all-cause stroke, major bleeding, minor bleeding, and acute kidney injury.

For the present analysis, patients at high surgical risk, defined as those with an STS score >8%, were excluded. Intermediate-to-low-risk patients were included irrespective of the type of prosthesis and categorized according to their age into two groups, i.e., ≥75 vs. <75 years.

### Statistical Analysis

Categorical variables were reported as frequencies and percentages and compared with the Chi-square test or Fisher’s test, as appropriate. A visual assessment of distribution was conducted for continuous variables, which were thereafter reported as mean (standard deviation—SD) or median (quartile 1–quartile 3 (Q1–Q3)) and compared by means of the Student’s *t*-test or Wilcoxon–Mann–Whitney test, as appropriate.

The cumulative, unadjusted frequencies of all-cause death in patients aging ≥75 vs. <75 years were obtained with the Kaplan–Meier method and compared through the log-rank test. Univariate and multivariable Cox regression analyses were run to obtain the predictors of the same outcome, selecting candidates’ variables on a clinical and statistical basis. Hazard ratios (HRs) along with 95% confidence intervals (CIs) were calculated. Given the time-dependent nature of the outcome, the predictive accuracy of age was assessed through a time-dependent area under the receiver operating characteristic curve (AUC). The optimal cutoff point of age for the prediction of all-cause mortality was also assessed using the Youden index estimator.

Finally, to account for the possible heterogeneity across the large population of the registry, sensitivity analyses were conducted on the primary and secondary outcomes by stratifying the population according to age quartiles.

Two-tailed *p*-values <0.05 were considered statistically significant. The analysis was conducted using “R” software (The R Foundation for Statistical Computing, Vienna, Austria, version 3.6.2).

## 3. Results

### 3.1. Baseline and Procedural Features

Among the 3389 patients initially enrolled in the registry, 411 were categorized as high surgical risk, and 293 were excluded due to the absence of follow-up data. Thus, the final population encompassed 2685 individuals (age < 75 years, *n* = 280; ≥75 years, *n* = 2405).

The baseline features are reported in Table 1. The median age was 82 (IQR 79–86) years in the whole population, with patients in the two arms being divided by almost a decade of age on average. Most patients were female, with a higher proportion of males in the <75 years group (45% vs. 35%, *p* = 0.001). Older patients had a lower BMI (26.7 (24–30) vs. 28.5 (24–33), *p* < 0.001), a higher frequency of previous pacemaker or implantable cardioverter defibrillator implantation (11% vs. 6%, *p* = 0.019), atrial fibrillation (AF, 33% vs. 23%, *p* = 0.010), and chronic kidney disease (CKD, 65% vs. 30%, *p* < 0.001). Contrary to this, smoking (19% vs. 7%, *p* < 0.001), diabetes (33% vs. 25%, *p* = 0.010), and chronic obstructive pulmonary disease (25% vs. 16%, *p* < 0.001) were more frequent in patients aging <75 years. The mean STS score was 3.60% (IQR 2.50–5.03) in the overall population, with low-risk patients being significantly more represented in the younger arm (72% vs. 55%, *p* < 0.001) and intermediate-risk patients in the older one (45% vs. 28%, *p* < 0.001).

No significant differences were noted with respect to most echocardiographic data, including mean aortic valve gradient and aortic valve area. Slight, yet statistically significant, differences in terms of left ventricular ejection fraction were present (60 (55–65) in the ≥75 years arm vs. 60 (54–65) in the <75 years arm, *p* = 0.006). CT scans revealed a higher prevalence of porcelain aorta in younger patients (16% vs. 9%, *p* < 0.001).

From a procedural standpoint (Table 2), the Evolut PRO model was predominantly implanted in the youngest arm (17% vs. 11%, *p* = 0.001), with no other significant difference between the two groups.

### 3.2. Clinical Outcomes

**Figure 1:**
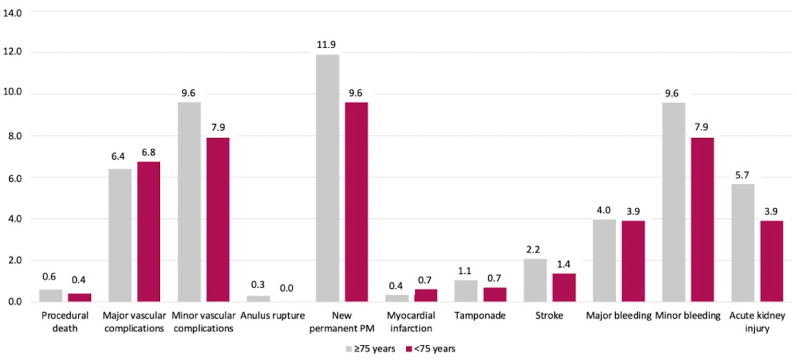
In-hospital events. Bars’ height indicates the percentage of adverse events in each arm. All *p* values were above 0.05. AKI: acute kidney injury; MI: myocardial infarction; PM: pacemaker.

As summarized in Figure 1, there were no significant differences across several in-hospital endpoints between the two groups.

**Figure 2:**
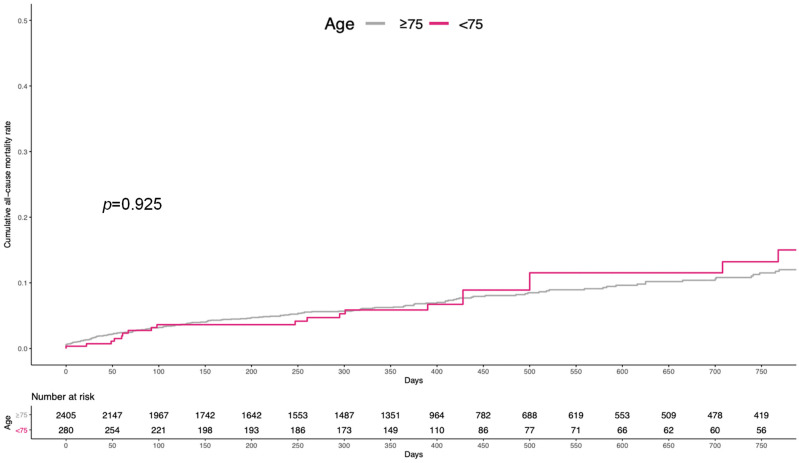
Kaplan–Meier curves for all-cause mortality.

Figure 2 depicts the cumulative incidence of all-cause mortality through a mean follow-up of 437 ± 381 days (86.5% of the population completed the 1-year follow-up). Overall, 198 (8.2%) and 23 (8.2%) patients died in the ≥75 and <75 years arm, respectively (log-rank *p* = 0.925). Likewise, the 1-year event rate was comparable with 124 (5.1%) and 13 (4.6%) deaths in the two arms, respectively, with no statistically significant difference (log-rank *p* = 0.707).

The results of the univariate and multivariable Cox regressions are reported in Table 3. In the univariate analysis, age was not associated with the risk of all-cause mortality, either when analyzed as a continuous covariate (HR 1.01, 95% CI 0.99–1.04, *p* = 0.294) or dichotomizing according to the prespecified cutoff of 75 years (HR 0.97, 95% CI 0.63–1.51, *p* = 0.924). New York Heart Association Class III or IV at presentation (HR 1.68, 95% CI 1.23–2.35, *p* = 0.001), CKD (HR 1.47, 95% CI 1.05–2.08, *p* = 0.026), atrial fibrillation (HR 1.40, 95% CI 1.06–1.84, *p* = 0.016), and the STS score (HR 1.12, 95% CI 1.03–1.21, *p* = 0.007) significantly predicted the occurrence of the primary outcome after adjusting for covariates.

The results of the sensitivity analysis according to the age quartiles are reported in the Supplementary Material. There was no significant difference regarding in-hospital outcomes across the different quartiles, except for the rate of a new pacemaker implantation, which was slightly higher in patients whose age was above the median (Supplementary Table S1). Supplementary Figure S1 shows the cumulative incidence of all-cause mortality in the four groups, with patients in the highest quartile experiencing a numerically higher event rate, without any statistically significant trend.

### 3.3. Accuracy of Age to Predict All-Cause Mortality

**Figure 3:**
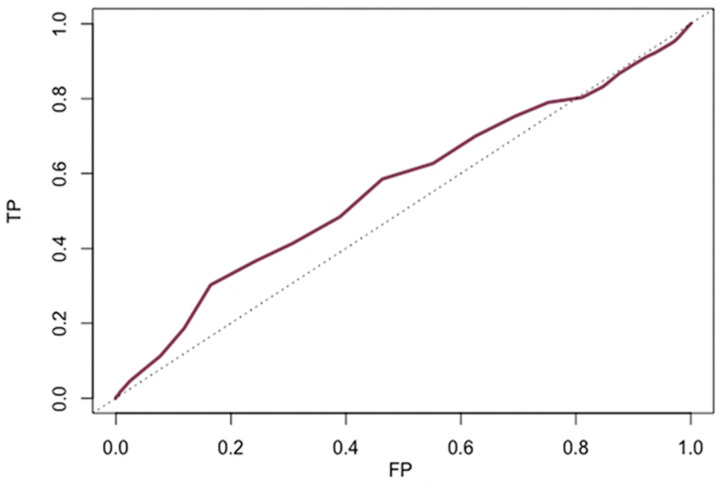
Time-to-event receiving operator characteristic (ROC) curve for 2-year all-cause mortality. AUC = 0.54. TP, true positive; FP, false positive.

The time-dependent RO curve showed low accuracy of age to predict either 1-year or 2-year all-cause deaths (both areas under the curve = 0.54) (Figure 3). The optimal age threshold to predict both 1-year (sensitivity 31%, specificity of 79%) and 2-year (sensitivity 32%, specificity of 78%) mortality was 85 years.

## 4. Discussion

Our extensive study delves deep into reevaluating the predominant emphasis on age as the primary determinant in transcatheter aortic valve implantation (TAVI) for severe aortic stenosis (AS). With a robust cohort of 2685 individuals, our analysis provides a comprehensive exploration of the intricate interplay between age, baseline characteristics, and clinical outcomes post-TAVI. In this large cohort of low-risk individuals treated with self-expandable devices, clinical outcomes were similar in patients in and out of the age cutoff recommended by the current ESC/EACTS guidelines, and age alone displayed a low accuracy in predicting all-cause mortality up to two years after transfemoral TAVI. The baseline characteristics uncovered a stark divergence in age distribution among cohorts, illustrating a median age of 82 years across the entire cohort. Notably, older patients exhibited a higher prevalence of comorbidities, including atrial fibrillation (33% vs. 23%, *p* = 0.010), chronic kidney disease (65% vs. 30%, *p* < 0.001), and a higher frequency of previous pacemaker/implantable cardioverter defibrillator implantation (11% vs. 6%, *p* = 0.019), emphasizing significant disparities in baseline profiles.

Previous studies have focused on the prognostic relevance of age in patients undergoing TAVI. An analysis of the Society of Thoracic Surgeons/American College of Cardiology Transcatheter Valve Therapy Registry, as well as a more recent report from the Swiss TAVI Registry found higher mortality rates in nonagenarians compared to younger patients, either during the first month or throughout the first year of follow-up [13]. Similar results were also reported by the Cerebrovascular Events in Patients Undergoing Transcatheter Aortic Valve Implantation (CENTER) collaboration [14]. These observations only emphasize the importance of patient selection among the very elderly, while the prognostic impact of age on most patients undergoing TAVI has been proven to be limited.

Contrary to conventional assumptions, our comprehensive data challenge the simplistic correlation between age, comorbidities, and adverse events post-TAVI. Despite notable differences in baseline characteristics, clinical outcomes did not significantly differ between patients aged below and above 75 years. This disparity underscores the intricate nature of risk assessment in TAVI candidacy, suggesting that age, in isolation, might inadequately capture the complex risk profiles among severe AS patients.

The nuanced insights derived from our data underscore the imperative need for a more comprehensive, holistic approach to risk stratification for TAVI. Beyond the age-related considerations, a multifaceted evaluation framework incorporating anatomical intricacies, procedural nuances, and a comprehensive comorbidity profile emerges as pivotal in refining risk assessment and optimizing treatment strategies in severe AS management.

Embracing a multifaceted evaluation framework becomes paramount in refining risk assessment and tailoring precision treatments in severe AS management, particularly within the realm of TAVI. The integration of anatomical assessments, procedural intricacies, and a comprehensive comorbidity profile holds substantial promise for optimizing patient outcomes. Our data-driven insights advocate for a paradigm shift towards personalized and optimized treatment strategies. This approach, integrating multifaceted patient factors beyond age, aligns with contemporary trends towards precision medicine, aiming to maximize patient outcomes through tailored treatment strategies [15,16].

Indeed, we have the first data from randomized controlled trials on TAVI vs. SAVR in intermediate- and low-risk settings. The patients showed no significant interaction according to the age subgroups for the rate of their primary endpoints. Most patients enrolled in these RCTs were substantially younger compared to earlier studies conducted on high-risk and inoperable populations, up to an average of less than 75 years in the Placement of Aortic Transcatheter Valves (PARTNER) 3 and Evolut surgical replacement and transcatheter aortic valve implantation in low-risk patients (Evolut low-risk) trials [2,4]. The extended follow-up data, particularly the 5-year analysis, presents an intricate evolution of TAVI outcomes. While the initial reports favored TAVI, longer-term observations prompt nuanced considerations. Notably, mortality data encompassing both cardiovascular and non-cardiovascular deaths reveal intriguing trends. Adjudication of cardiovascular deaths with stringent criteria in the PARTNER 3 trial showed nuanced rates at 5 years, underscoring the need for cautious interpretation. Additionally, the apparent attenuation of differences in primary endpoint rates between TAVI and SAVR warrants attention. While several secondary endpoints favor TAVI, factors such as residual aortic regurgitation and valve thrombosis lean towards SAVR, emphasizing the complex landscape of technological advancements and therapeutic evolution [17]. On the other side, the Evolut low-risk 4-year examination compares TAVR versus SAVR in low-risk aortic stenosis patients, revealing a 26% lower risk of death or disabling stroke with TAVR. Over time, TAVR’s advantage widens, with a 3.4% difference at 4 years. Notably, TAVR showcases improved hemodynamics but a higher rate of pacemaker implantation. This highlights the need for a decade-long follow-up to comprehensively gauge valve durability and performance in this population [18].

These studies provided the backbone for either the European guidelines, which substantially encourage TAVI in all patients of 75 years or beyond, or the US guidelines, which further pushed the recommendation by advocating TAVI as a possible alternative to SAVR in all patients between 65 and 80 years of age.

For younger patients choosing surgical aortic valve replacement (SAVR), options for dealing with future valve issues include TAV-in-SAV or redo SAVR. For those opting for transcatheter aortic valve replacement (TAVR), potential solutions for valve failure encompass TAVR explant with SAVR or TAV-in-TAV. Understanding these options before the initial intervention is crucial, as it shapes future treatment pathways [9,10,11,12,13].

With the recent approval of low-risk TAVR, managing a SAVR-first strategy is more established. While TAV-in-SAV shows promising short-term outcomes over a redo SAVR, concerns about its durability exist. The redo SAVR involves higher risks but offers advantages like lower postoperative gradients and less leakage. Ongoing research is exploring reintervention possibilities in a TAVR-first strategy [16,17,18,19,20,21,22].

Contrarily to older patients, those in their 70s or 60s have been substantially underrepresented in RCTs published so far, with most evidence nowadays available deriving from observational studies. In the German Quality Assurance Registry on Aortic Valve Replacement (AQUA) [19], for instance, SAVR and transfemoral TAVI were compared among patients <75 years. After propensity matching, in-hospital outcomes did not differ between the two cohorts, except for a higher rate of permanent pacemaker implantation in TAVI patients and a higher rate of delirium in SAVR patients. Later, Witberg and colleagues [20] compared patients <70 and ≥70 years who had undergone TAVI, showing a similar incidence of in-hospital adverse events and a similar rate of all-cause mortality up to 5 years. Taken together, these observations further support the substantial equipoise of the surgical and transcatheter approaches in the youngest candidates as well, with a consistent benefit of TAVI in terms of hard clinical outcomes across different ages. The present study further expands this literature, confirming a comparable safety–efficacy profile of TAVI in patients at the two extremes of the prespecified age threshold. Differently from the previously published data, however, we focused on a guideline-recommended cutoff, which makes our study of immediate practical relevance by providing clinicians and interventionalists with a tool to enhance guideline understanding and critically appraise their content.

Although our study did not find any relevant impact of age on clinical outcomes following transfemoral TAVI, a high number of factors tightly intertwined with patients’ age, unfortunately not fully captured by our data, should be taken into account. First and foremost, as shown by previous studies performed on patients treated with SAVR, young age is among the drivers of structural valve deterioration [21]. Notwithstanding the similarities between the prostheses adopted for TAVI and SAVR, it remains currently uncertain whether the long-term performance of the first might reproduce that of surgical valves. Moreover, even in the era of alignment techniques, current devices for TAVI carry the risk of making coronary re-access harder compared to surgical prostheses [22]. Therefore, they could potentially hamper future percutaneous coronary revascularizations, which is of particular interest to young patients [23].

Hence, the choice of the type of intervention should be weighted on several factors, among which age remains, without doubt, essential. Substantial heterogeneity in terms of treatment outcomes is unlikely to be exclusively predictable by age itself. Harmonizing technical and clinical aspects as well as patients’ preferences, as encouraged by the current guidelines, will remain a must in the near future. In the pursuit of lifelong management of aortic treatment, the evolving dynamics underscore the necessity for continued exploration, embracing evolving technologies, and collaborative efforts. The uncharted territory necessitates a collaborative approach between heart teams, scientific researchers, and surgical entities to navigate this realm of evolving strategies, affirming that the game for aortic treatment’s lifelong management remains wide open.

### Limitations

The retrospective and non-randomized design is an inherent and key limitation of the present study, preventing us from excluding the influence of confounders and limiting our capability to explore all the potential effect modifiers. Missing data (13.5%) might have influenced the reliability of the analysis. We would have aimed at performing further analyses to explore the age cutoffs supported by the US guidelines; unfortunately, this was substantially impossible due to the very low number of patients aged less than 65 years. Furthermore, patients included in the dataset may not be representative of those implanted with balloon-expandable valves, which were not enrolled in the HORSE registry. Finally, due to the lack of long-term clinical and echocardiographic follow-up, we cannot estimate the impact of structural valve deterioration.

## 5. Conclusions

Careful, case-by-case evaluation of patient features and preferences remains a must in severe AS. Age-related issues exist and should be considered when choosing the best treatment strategy. However, the age cutoff advocated by contemporary guidelines does not stratify the occurrence of hard adverse events, either during the hospital stay or through a mid-length follow-up.

## Figures and Tables

**Table 1:** Baseline features.

	All N = 2685	≥75 Years N = 2405	<75 Years N = 280	*p*-Value
**Clinical characteristics**				
Age, years	82 (79–86)	83 (80–86)	72 (69–73)	<0.001
Male sex	960 (36)	835 (35)	125 (45)	0.001
Body mass index, kg/m^2^	26.9 (24–31)	26.7 (24–30)	28.5 (24–33)	<0.001
Hypertension	2334 (87)	2092 (87)	242 (86)	0.813
Dyslipidemia	1035 (52)	925 (52)	110 (51)	0.933
Diabetes	705 (26)	613 (25)	92 (33)	0.010
Smoke	228 (15)	176 (7)	52 (19)	<0.001
Prior myocardial infarction	482 (18)	441 (18)	41 (15)	0.144
Prior percutaneous coronary intervention	731 (27)	656 (27)	75 (27)	0.905
Prior stroke	287 (11)	261 (11)	26 (9)	0.478
Chronic obstructive pulmonary disease	450 (17)	381 (16)	69 (25)	<0.001
PM or ICD	276 (10)	259 (11)	17 (6)	0.019
Atrial fibrillation	2334 (87)	795 (33)	65 (23)	0.010
Chronic kidney disease	1645 (61)	1562 (65)	83 (30)	<0.001
Baseline creatinine, mg/dL	1.10 (0.61)	1.09 (0.55)	1.19 (0.98)	0.010
Peripheral arterial disease	333 (12)	297 (12)	36 (13)	0.890
NYHA III-IV	1787 (67)	1607 (67)	180 (64)	0.390
STS score, %	3.60 (2.50–5.03)	3.73 (2.60–5.10)	2.55 (1.75 4.10)	<0.001
Low risk	1520 (57)	1318 (55)	202 (72)	<0.001
Intermediate risk	1165 (43)	1087 (45)	78 (28)	<0.001
**Echocardiographic data**				
Mean aortic valve gradient, mmHg	45.1 (16.2)	45.2 (16.3)	44.9 (15.6)	0.821
Aortic valve area, mm^2^	0.76 (2.73)	0.77 (2.87)	0.73 (0.19)	0.850
Left ventricular ejection fraction, %	57 (55–60)	55 (52–58)	60 (55–65)	0.006
Moderate-severe aortic regurgitation	56 (3)	51 (3)	5 (2)	0.966
**MDCT data**				
Perimeter, mm	70 (26–76)	70 (26–76)	71 (26–77)	0.367
Moderate–severe aortic valve calcification	1173 (44)	1055 (44)	118 (42)	0.971
Moderate/severe LVOT calcification	446 (16)	408 (17)	38 (14)	0.224
Porcelain aorta	271 (10)	225 (9)	46 (16)	<0.001

ICD: implantable cardioverter defibrillator; LVOT: left ventricular outflow tract; MDCT: multidetector computerized tomography; NYHA: New York Heart Association; PM: pacemaker; STS: The Society of Thoracic Surgeons.

**Table 2:** Procedural data.

	All N = 2685	≥75 Years N = 2405	<75 Years N = 280	*p*-Value
Predilatation	1452 (54)	1309 (54)	143 (51)	0.309
Valve type				
Evolute R	1068 (40)	963 (40)	105 (38)	0.449
Evolute PRO	301 (11)	253 (11)	48 (17)	0.001
ACURATE neo	1316 (49)	1189 (49)	127 (45)	0.219
Valve size, mm				0.025
<23	423 (16)	381 (16)	42 (15)	
23–26	1021 (38)	933 (39)	88 (31)	
≥27	1241 (46)	1091 (45)	150 (54)	
Post-dilatation	892 (33)	788 (33)	104 (37)	0.153
Contrast dose, ml	110 (80–160)	110 (80–160)	110 (80–150)	0.612
Fluoroscopy time, minutes	16 (10–24)	16 (10–24)	15 (10–23)	0.244

**Table 3:** Predictors of mortality.

	Univariable	Multivariable		
Variable	HR (95% CI)	*p*-Value	HR (95% CI)	*p*-Value
Age (1-year increase)	1.01 (0.99–1.04)	0.294		
Male sex	1.14 (0.87–1.50)	0.336		
Prior MI	1.19 (0.84–1.68)	0.323		
Diabetes	1.05 (0.78–1.42)	0.746		
NYHA III-IV	1.92 (1.40–2.63)	**<0.001**	1.68 (1.23–2.35)	**0.001**
COPD	1.12 (0.80–1.56)	0.502		
Prior stroke	1.02 (0.67–1.57)	0.928		
PAD	1.37 (0.96-.95)	0.082		
CKD	1.75 (1.30–2.37)	**<0.001**	1.47 (1.05–2.08)	**0.026**
AF	1.48 (1.13–1.93)	**0.004**	1.40 (1.06–1.84)	**0.016**
Baseline creatinine, mg/dL (1-unit increase)	1.28 (1.09–1.50)	**0.002**	1.12 (0.91–1.38)	0.272
Permanent PM or ICD	1.20 (0.81–1.79)	0.359		
STS score (1% increase)	1.16 (1.08–1.26)	**<0.001**	1.12 (1.03–1.21)	**0.007**
EF (1% increase)	0.99 (0.98–1.00)	0.229		
Predilatation	1.06 (0.82–1.39)	0.648		
Post-dilatation	1.02 (0.77–1.35)	0.874		
Valve size, mm (vs. <23)				
23–26	0.99 (0.65–1.53)	0.993		
≥27	1.28 (0.86–1.94)	0.227		

Bold: significant results.

## Data Availability

Data is unavailable due to privacy and ethical restrictions.

## Supplementary Materials

The following supporting information can be downloaded at https://www.mdpi.com/article/10.3390/jcdd11010033/s1. Table S1: In-hospital outcomes according to age quartiles; Figure S1: All-cause mortality according to age quartiles. Table S2: Predictors of mortality, including age as a factor, beyond its non-significance at the univariate analysis.
