# Supplementary material for: Comparison of Mid-Term Prognosis in Intermediate-to-Low-Risk Contemporary Population with Guidelines-Oriented Age Cutoff
Source: J Cardiovasc Dev Dis. 2024 Jan 22;11(1):33. doi: 10.3390/jcdd11010033 (PMC10816002; doi:10.3390/jcdd11010033)
Supplement: Supplementary file 1 [file jcdd-11-00033-s001.zip › jcdd-2773947-supplementary.pdf]

Supplementary Material

Comparison of Mid-Term Prognosis in Intermediate-to-Low-Risk Contemporary Population with Guide-lines-Oriented Age Cutoff.

I. STUDY DEVICES

II. PARTICIPATING CENTERS

III. LIST OF COVARIATES USED FOR THE INVERSE PROBABILITY WEIGHTING ANALYSIS

IV. SUPPLEMENTARY TABLES AND FIGURES

I. STUDY DEVICES

The Evolut R System (Medtronic, Inc., Minneapolis, Minnesota) comprises the Evolut R valve and the EnVeo R Delivery catheter system (DCS) with the InLine sheath. The porcine pericardial tissue trileaflet valve is sutured in a supra-annular position on a compressible and self-expandable nitinol frame. The EnVeo R DCS enables the valve to be fully repositionable and recapturable before full release. The built-in InLine sheath allows for the whole system to be inserted into a patient without the need for a separate access sheath, equivalent to the outer diameter of a 14-F sheath [1]. The Evolut R valve is currently available in 23, 26, 2m9 and 34 mm sizes. The Evolut PRO System (Medtronic, Inc., Minneapolis, Minnesota) is delivered transfemorally using a dedicated sheathless delivery system with an outer diameter of a 16-F sheath. The device has the same shape and properties as the second-generation Evolute R version, except for an outer adjunctive pericardial skirt to enhance annular sealing. The Evolute PRO valve is currently available in 23-, 26-, and 29-mm sizes.

The Acurate NEO bioprosthesis is composed of porcine pericardial valve leaflets mounted on a self-expanding nitinol frame in a supra-annular position, with a pericardial sealing skirt on the outer and inner surfaces of the stent body. The system is implanted using a dedicated transfemoral delivery system inserted through a 20-F sheath. At the top of the valve, there are three flexible and repositionable stabilization arches, ensuring coaxial alignment. The upper crown ensures stable positioning and anchoring of the native leaflets, which theoretically can reduce the risk of coronary obstruction and paravalvular leaks [2]. The device is currently available in sizes small, medium, and large.

II. PARTICIPATING CENTERS

1. Interventional Cardiology Unit, Maria Cecilia Hospital, GCM Care and Research

2. Department of Cardiology, Cardiovascular Institute, Hospital Clínico San Carlos, Madrid, Spain

3. The Heart Center, Rigshospitalet, Copenhagen

4. Department of Cardiology and Cardiac Surgery, Kerckhoff Heart and Lung Center, Bad Nauheim, Germany

5. Department of Cardiovascular Surgery, University Heart Center, Hamburg, Germany

6. Department of Cardiology, C.A.S.T. Policlinic G. Rodolico Hospital, University of Catania, Catania, Italy

7. Cardio Center, Humanitas Research Hospital, Rozzano-Milan, Italy

8. Division of Cardiology, Pulmonology, and Vascular Medicine, University Hospital Düsseldorf, Düsseldorf, Germany

9. Department of Cardiology, IRCCS Policlinico San Donato, Milan, Italy

10. Heart Valve Clinic, University Hospital of Zürich, Zürich, Switzerland

11. Quebec Heart Institute, Laval Hospital, Quebec, Canada

12. Department of Cardiology, Hospital of León, León, Spain

13. Department of Cardiology, Galway University Hospitals, Galway, Ireland

14. Clinic Cardiovascular Institute, University Hospital Clinic, IDIBAPS, Barcelona, Spain

15. "Evangelismos" Hospital, National and Kapodistrian University of Athens, Greece

16. Interventional Cardiology Unit, Ospedali Riuniti di Ancona, Italy

III. LIST OF COVARIATES INCLUDED IN THE INVERSE PROBABILITY WEIGHTING ANALYSIS

Age
Diabetes
Body mass index
Hypertension
Aortic regurgitation □ _moderate
Male
NYHA III or IV functional class
Aortic valve calcification □ _moderate
Left ventricle outflow tract calcification □ _moderate
Low gradient aortic stenosis
Atrial fibrillation
Chronic obstructive pulmonary artery disease
Peripheral artery disease
Porcelain aorta
Previous cardiac surgery
Previous myocardial infarction
Previous percutaneous coronary interventions
Previous pacemaker/defibrillators
Previous stroke
Ejection fraction
Transcatheter prosthesis size
Annulus perimeter size
The Society of Thoracic Surgeons score

IV. SUPPLEMENTARY TABLES AND FIGURES

Supplementary Table S1. In-hospital outcomes according to age quartiles

**Outcome**	**I quartile (N=679)**	**II quartile (N=559)**	**III quartile (N=799)**	**IV quartile (N=648)**	**P value**
Procedural death	3 (0.4)	2 (0.4)	6 (0.8)	5 (0.8)	0.726
Vascular complications					
Major	38 (5.6)	34 (6.1)	57 (7.1)	44 (6.8)	0.638
Minor	65 (9.6)	55 (9.8)	76 (9.5)	58 (9.0)	0.963
Anulus rupture	1 (0.1)	1 (0.2)	3 (0.4)	2 (0.3)	0.848
New permanent PM	64 (9.4)	56 (10)	106 (13.3)	86 (13.3)	0.036
Myocardial infarction	4 (0.6)	2 (0.4)	2 (0.3)	3 (0.5)	0.787
Tamponade	3 (0.4)	6 (1.1)	15 (1.9)	5 (0.8)	0.069
Stroke	12 (1.8)	12 (2.1)	20 (2.5)	13 (2.0)	0.815
Bleeding					
Major	23 (3.4)	20 (3.6)	37 (4.6)	28 (4.3)	0.525
Minor	72 (10.6)	54 (9.7)	70 (8.8)	57 (8.9)	0.547
AKI	32 (4.7)	28 (5.0)	55 (6.9)	34 (5.2)	0.677

Supplementary Figure S1. All-cause mortality according to age quartiles

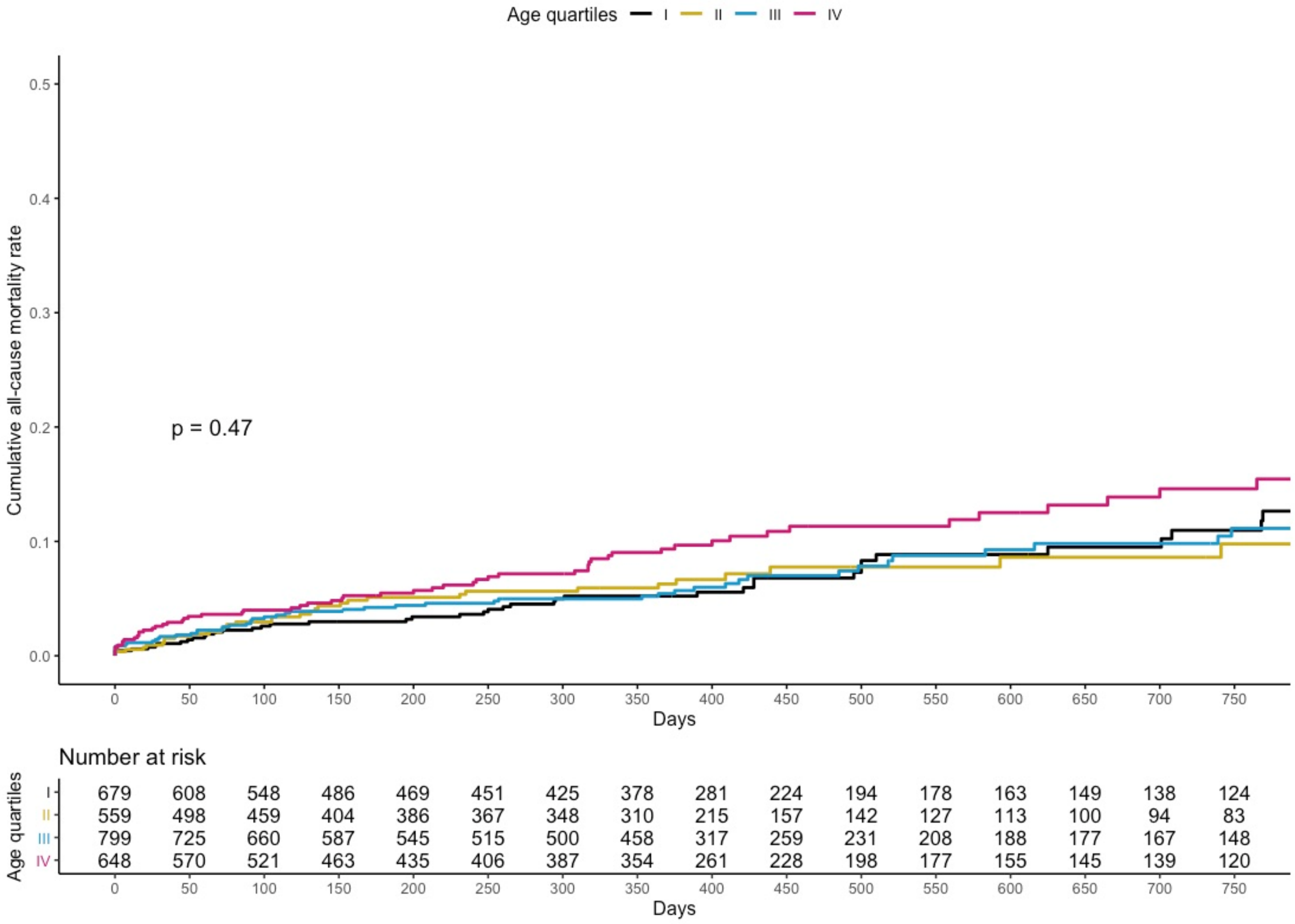

Table S2. Predictors of mortality, including age as a factor, beyond its non-significance at the univariate analysis.

	**Univariable**		**Multivariable**	
**Variable**	**HR (95% CI)**	**p-value**	**HR (95% CI)**	***p*-value**
Age (1-year increase)	1.01 (0.99-1.04)	0.294	1.00 (0.97-1.01)	0.428
Male sex	1.14 (0.87-1.50)	0.336		
Prior MI	1.19 (0.84-1.68)	0.323		
Diabetes	1.05 (0.78-1.42)	0.746		
NYHA III-IV	1.92 (1.40-2.63)	**<0.001**	1.65 (1.21-2.29)	**0.001**
COPD	1.12 (0.80-1.56)	0.502		
Prior stroke	1.02 (0.67-1.57)	0.928		
PAD	1.37 (0.96-.95)	0.082		
CKD	1.75 (1.30-2.37)	**<0.001**	1.32 (1.05-1.87)	**0.031**
AF	1.48 (1.13-1.93)	**0.004**	1.24 (1.02-1.7)	**0.018**
Baseline creatinine, mg/dl (1-unit increase)	1.28 (1.09-1.50)	**0.002**	1.12 (0.91-1.38)	0.272
Permanent PM or ICD	1.20 (0.81-1.79)	0.359		
STS score (1% increase)	1.16 (1.08-1.26)	**<0.001**	1.09 (1.02-1.18)	**0.011**
EF (1% increase)	0.99 (0.98-1.00)	0.229		
Predilatation	1.06 (0.82-1.39)	0.648		
Post-dilatation	1.02 (0.77-1.35)	0.874		
Valve size, mm (vs. <23)				
23-26	0.99 (0.65-1.53)	0.993		
≥27	1.28 (0.86-1.94)	0.227		

References:

1. Laricchia, A.; Cereda, A.; Lucreziotti, S.; Sticchi, A.; Regazzoli, D.; Reimers, B.; Colombo, A.; Latib, A.; Mangieri, A. Expanding Our Horizons for the Use of Transcatheter Self-Expanding Valves: What Does the Future Hold? Expert Rev. Cardiovasc. Ther. 2022, 20, 497–501. https://doi.org/10.1080/14779072.2022.2085688.
2. Mack, M.J.; Leon, M.B.; Thourani, V.H.; Pibarot, P.; Hahn, R.T.; Genereux, P.; Kodali, S.K.; Kapadia, S.R.; Cohen, D.J.; Pocock, S.J.; et al. Transcatheter Aortic-Valve Replacement in Low-Risk Patients at Five Years. N. Engl. J. Med. 2023, 389, 1949–1960. https://doi.org/10.1056/NEJMOA2307447.
